# NicoLase—An open-source diode laser combiner, fiber launch, and sequencing controller for fluorescence microscopy

**DOI:** 10.1371/journal.pone.0173879

**Published:** 2017-03-16

**Authors:** Philip R. Nicovich, James Walsh, Till Böcking, Katharina Gaus

**Affiliations:** 1 ARC Centre of Excellence in Advanced Molecular Imaging, University of New South Wales, Sydney, New South Wales, Australia; 2 EMBL Australia Node in Single Molecule Science, School of Medical Sciences, University of New South Wales, Sydney, New South Wales, Australia; Cornell University, UNITED STATES

## Abstract

Modern fluorescence microscopy requires software-controlled illumination sources with high power across a wide range of wavelengths. Diode lasers meet the power requirements and combining multiple units into a single fiber launch expands their capability across the required spectral range. We present the NicoLase, an open-source diode laser combiner, fiber launch, and software sequence controller for fluorescence microscopy and super-resolution microscopy applications. Two configurations are described, giving four or six output wavelengths and one or two single-mode fiber outputs, with all CAD files, machinist drawings, and controller source code openly available.

## Introduction

Multiple laser lines are essential for a modern fluorescence microscope. Rarely can a system produce publication-appropriate results without at least a second illumination wavelength. Diode laser units are convenient for fluorescence microscopy and super-resolution microscopy due to their high power and ease of use. While somewhat more expensive than a noble gas mix laser plus an acousto-optical tunable filter [1], the long life, ease of use, and expanded wavelength repertoire of diode lasers have pushed the other options strongly out of favor [2]. Multiple makers provide continuous wave (CW) diode laser product lines with a dozen or more wavelengths from the ultraviolet through mid-infrared with powers from tens to a few hundred milliwatts [3] With the addition of direct modulation available on nearly all modern units there’s no need for an acousto-optical tunable filter or other power modulation control in addition to the diodes themselves [3].

Software and trigger control of the laser sources is strongly desired. Microscopy cameras often have a dead time between image captures when laser illumination is (almost always) undesired, and at the very least, it’s convenient to have a laser that shuts off when the acquisition is not actively running. As a further step, it’s often necessary to alternate or otherwise sequence excitation sources for certain experiments. For example, in alternating laser excitation fluorescence energy transfer (ALEX FRET) measurements, the acceptor and donor in the FRET pair are each directly excited in an alternating sequence, imaging with two emission channels at each frame [4]. Photoactivation with subsequent imaging experiments can use a single activating exposure (*i.e*. a pulse of 405 nm light), followed by many excitation exposures for imaging (*i.e*. 488 nm for photoactivated green fluorescent protein) [5]. Multi-color imaging by exciting with different wavelengths in turn across multiple acquisition frames. An ideal excitation system would be able to accomplish any of these approaches programmatically given a common set of hardware with output and switching synchronized to the imaging camera.

The excitation lasers can be free-space coupled into the microscope system, bouncing the beam between mirrors from the laser aperture to the back of the objective, but coupling into a single-mode fiber provides extra flexibility in system configuration [6]. This means the location of the lasers and their peripherals can be away from the remainder of the optical system. In addition, the fiber acts as a spatial filter, yielding enhanced beam quality and makes precise positioning of the beam, such as is what is required for total internal fluorescence (TIRF) microscopy [7], much more straightforward. Coupling to a single-mode fiber sets a high bar in terms of alignment of the multiple lasers into a combined output. The precision for free-space or multi-mode fiber coupling is comparatively relaxed. A system that can effectively align into a single-mode fiber can do all three should the application require it.

There are commercial options to achieve nearly all of these criteria. The presented system has a final cost that is 2/3 to 1/2 of that of commercial instruments with comparable performance and can be easily customized in terms of size, number of lines, and final configuration. Lab-built assemblies for laser combining are also common, but by sharing these implementations the hope is that downstream users can take advantage of these materials without having to reinvent the fiber launch, so to speak. In the same vein as projects such as OpenSPIM [8], this provides a starting point for others who are encouraged to share their own improvements and modifications.

**Fig 1:**
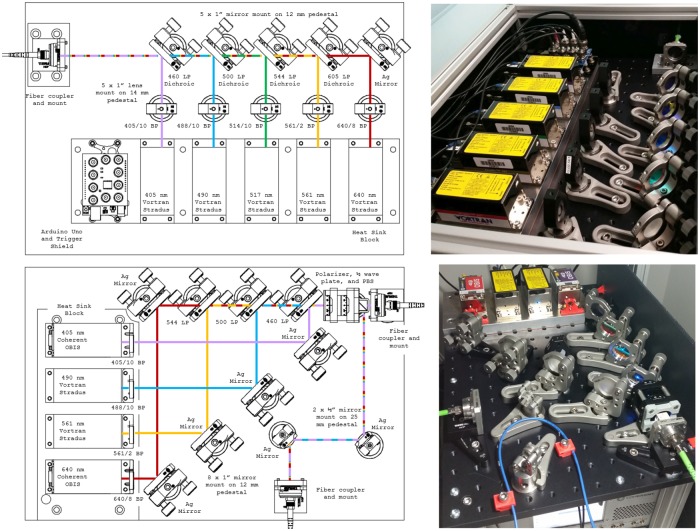
NicoLase schematics and implementations. Top—NicoLase 1500, showing 5 Vortran Stradus lasers combining to a single fiber output with one mirror per diode laser. The Arduino Uno with NicoLase shield sits in the sixth laser mounting location, though an additional diode could fit in this configuration. Bottom—NicoLase 2400, featuring four lasers, with two mirrors each, and output coupled split between two optical fibers. An Arduino Uno-based controller sits on the optical table below. The dual output hardware is described in section **One Fiber or Two?**.

Two generations of this project are presented. The first, referred to as the NicoLase 1500, is a single-mirror coupling system coupling five (expandable to six) diode lasers into a single-mode fiber output. The latter, the NicoLase 2400, is a two-mirror system with four diode laser lines with output power tunable between two single-mode fiber outputs. These share the same footprint, many of the same components, and the same control software and PC interface. Schematics and pictures of both designs appear in Fig 1. Details of the hardware, optics, controller, software, and performance are detailed in the subsequent sections.

All design files, bill of materials, software source code, and machine shop diagrams are included in the author’s GitHub repository, available at https://github.com/PRNicovich/NicoLase. This repository is continually maintained including updates for new iterations of the NicoLase project. Users are encouraged to generate a fork of this repository for their use and upload any of their own updates and modifications.

## Hardware

### Lasers

Any chosen laser diode unit needs to have the following characteristics: a 100 mm x 50 mm footprint or smaller; 19.00 mm or 3/4” (19.05 mm) beam height, or can be brought to this height with spacers/lifts; digital modulation via TTL pulse; and software control of laser power via USB or RS-232 (for add-on plug-in control). These qualifications have become a standard across the laser diode industry, so finding alternative products to those presented should not present any undue difficulty.

The NicoLase is built primarily around Vortran Stradus laser diode units. These are a standardized size for each line, reasonably priced, and produce a large amount of power for their size and cost. Nearly all produce enough power to achieve final intensities of a few kW/cm^2^ over a field of few tens of micrometers on a side. These units can be digitally modulated at a high rate (commonly up to 2 MHz) and potentially analog modulated if desired. Though not tested specifically for this application, a survey of products from major manufacturers confirms that those from Spectra-Physics (Excelsior), Oxxius (Laser Boxx), and Omikron (LuxX) are also compatible.

A straightforward hardware modification allows one to use Coherent OBIS heads in place of some or all of the Vortran units. Control is nearly identical and aside from repositioning of the mounting screw holes, these are drop-in replacements for the Vortran units. A similar and minor modification may be required for diode laser units from other makers.

### Heatsinks and mounting

All optics are chosen for a 1.5” (39.0 mm) center height. Mirrors and dichroic mirrors are mounted in Thorlabs Polaris 1” mounts. The single-mirror system requires 3-adjuster mounts, but either 2- or 3- adjuster mounts can be used for the two-mirror system. Mounds with hex adjusters rather than knobs allow more clearance between mounts (or more mounts in the same space) but at the cost of ease of adjustment. Clearances are minimally sufficient for mounts with knob adjusters throughout.

Mirrors and other optics atop standard Thorlabs pedestals except for the fiber coupler itself. This needs a custom pedestal to be machined, but this is a straightforward piece of turning. The original 1500 design includes a flat block for this mount while the 2400 pedestal is the preferred design. Pedestal clamps are standard Thorlabs parts, though lower-profile clamps are advantageous for some positions.

Excitation filters can be mounted in standard lens mounts, or options for 3D printed mounts for 1/2” filters designed for the NicoLase are available in the author’s 3D printing repository (https://github.com/PRNicovich/3D-Printed-Optics-Lab-Parts). A mount to tie the Arduino to the optical table or a spare slot in the heat sink block is given in the same repository. Estimated costs for printing these parts through an online supplier (shapeways, www.shapeways.com) is $5–8 for the filter mount and $9–11 for the Arduino mount, depending on color and material chosen.

The following criteria apply when choosing the mounting strategy for the laser units: diode laser units require a heat sink for proper operation; in this design the laser beam outputs need to be at 39.0 mm above the breadboard surface to correctly intersect with the steering and coupling optics; and of course lasers have to be solidly attached to a mounting point for steady alignment.

To address these issues, a heat sink and mounting block was machined. Three dimensions must be held to strict tolerances for this part. The top must be as flat as possible (0.1 mm specified) with no scratches or machine marks to hinder interfacing with the bottom of the laser diode for optimal heat transfer. The top and bottom faces must be parallel (0.1 mm specified) so the beams are at the same height. The total height must be the specified thickness (0.2 mm specified). Finally the mounting holes must be in the proper location, which will depend on the laser product chosen (for a Vortran Stradus, this is M4 x 0.7 threaded holes in a 90 mm x 30 mm rectangle; 0.5 mm position tolerance specified).

Keeping the top flat can be done by using a piece of ground stock and machining other faces. Keeping the top and bottom flat to that tolerance should be straightforward for a standard vertical mill. And a trick can be employed for the total height. This is because there are much larger tolerances for the roughness and finish of the bottom of the part and we can therefore add shims of sheet stock (with clearance holes cut) to bring the total assembly to the desired height. The block can be machined on the bottom face until the thickness is short of the target, then a piece (or pieces) of sheet stock selected to bring it up to the final thickness. Sheet stock being available in nearly a continuous range of thicknesses makes this much simpler and easier to meet the necessary tolerance than achieving the right thickness with machining only.

The heat sink block must be made of either aluminum or copper. Steel, especially stainless, does not have a sufficient heat transfer rate to keep the diodes from overheating [9]. The presented top blocks were made from aluminum and the shims from copper sheet, which works very well. The high tolerance flat interface between the diode unit and heat sink does a sufficient job at heat transfer and heat sink compound is not recommended [10].

The parts can be manufactured using manual machining services or by CNC machining. For reference, CNC machining through Proto Labs (www.protolabs.com) quotes $1,267 for the 1500 block, $464 for the 2400 block, and $168 for the fiber launch pedestal, each in single quantity in 6061 aluminum. Manual machining costs will depend on the local supplier, but may be more economical when those sources are available.

Enclosures keep the lasers away from the eyes of users and the optics protected from bumps and dust. Drawings and pictures of enclosures for the assembled units are provided. The 1500 enclosure, made from T-slot aluminum and plastic panels (sourced from 80/20) is ‘facility-ready’ and is appropriate for multi-user environments (est cost $550). A bill of materials and associated measured drawings sufficient to recreate the enclosure from a local supplier are included in the repository. The implemented 2400 enclosure is made of corrugated plastic sheeting and secured with magnets and 3D printed magnet clips (files available at the author’s GitHub repository; ~$7–13 per 16 clips to print, depending on style). In both cases the laser power supplies are on the optical table with the breadboard and lasers above, but there’s no reason the whole assembly couldn’t be moved to a shelf off of the optical table if space is at a premium.

Technical drawings for all machined components are included in the NicoLase GitHub repository. These drawings were sufficient for the University’s machine shop to complete satisfactory work with no additional consultation.

### Optics

Optics are relatively straightforward—three or four dichroic mirrors, optional filters, and a fiber coupler. The units presented were built with Thorlabs components and these parts are provided as reference, but similar products from any other maker should be compatible.

**Fig 2:**
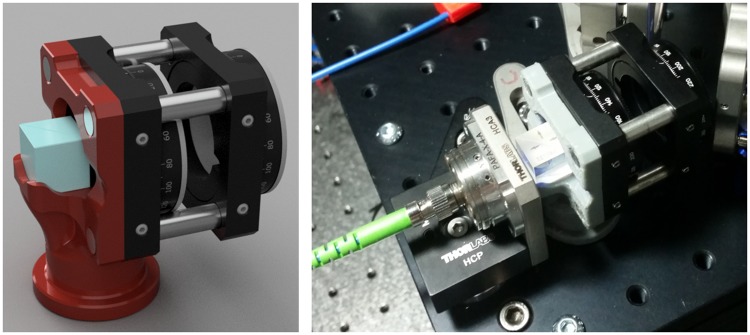
Dual output mount. Left—Render of mount design. A 3D-printed mount (red) holds a 12.5 mm polarizing beamsplitter cube (blue) and four 6 mm rods (silver). These rods support two 30 mm cage rotation mounts, the rightmost with a linear polarizer and leftmost a half-wave plate. Right—installed mount (gray) in place in the NicoLase 2400 immediately upstream of the transmitted fiber coupler.

Dichroic mirrors are used to combine lasers into a single multi-wavelength beam. A mix of Chroma and Semrock filters were used and part numbers given in the repository and nominal specifications in Fig 2. All are standard 1” round long-pass filters costing a few hundred USD each. Which ones are used depends on which wavelengths are being combined, though examples for commonly-used laser wavelength combinations used are given in the repository. A filter must have a cut-on wavelength that reflects the beam being folded in and transmits all others upstream. Picking a cut-on wavelength that falls between adjacent laser lines usually works well, but the provided spectra should be consulted to confirm there are not odd peaks or dips in the filter spectrum.

Diode lasers often require an emission clean-up filter to block odd long-wavelength emission from the laser head. This seems especially prevalent with blue (470–490 nm) diode units. At the same time, diode heads are ordered by nominal wavelength, but the actual wavelength of the supplied unit may vary 3–5 nm from this nominal value. This means that when choosing emission clean-up filters, one cannot necessarily order a laser line filter for the nominal wavelength and have it work for a given diode. For example, the shown ‘488’ nm diodes actually emit at 490 nm, and a 488 nm laser line clean-up filter (Semrock LL01-488-12.5) blocks nearly half of the emitted power. A 485/10 band-pass filter does a much better job transmitting the desired diode output and blocks the offending green emission. This means one must double-check the actual emitted wavelength (should be specified by the vendor in the factory testing documentation) when choosing an emission filter or if emitted wavelength is critical.

For the mirrors and associated mounts, 1” optics were chosen. This was primarily motivated by the availability of long-pass dichroic mirrors in a standard 25 mm round size, while smaller 12.5 mm or 0.5” long-pass mirrors are less common. The 1” mounts are the same cost as the 0.5” mounts and do provide a larger clear aperture when mounted at 45 to the optical path. Bandpass filters for laser clean-up are often available in 12.5 mm round size, often at significant savings relative to the 25 mm round option, and the smaller filters were used where possible.

A Thorlabs PAFA-X-F-A Achromatic FiberPort FC/APC coupler is used to couple the combined laser beams into a polarization-maintaining FC/APC patch cable (Thorlabs P3-405BPM-FC-2). The FiberPorts are somewhat expensive (~$500 USD), but these units have proven to be stable and relatively achromatic. The quoted item has a clear aperture of 1.8 mm at the input lens, which defines the upper limit for laser beam size that can be efficiently coupled into the fiber. Thankfully most diode laser beam sizes are smaller than this, with $\frac{1}{e^{2}}$ diameters of 0.6 mm to 1.1 mm being common.

The selected fiber was chosen on the basis of an acceptable operating wavelength range and low insertion loss; if substituting it is important to ensure the fiber will transmit the laser wavelengths used and angle-polished connectors are good for increased coupling efficiency. A polarization-maintaining fiber is not strictly necessary if the excitation mode does not require an input of controlled polarization. In our hands 30–60% coupling efficiency across all lines is achievable without too much struggle on this configuration.

A bill of materials for optical components as well as an assembly and alignment guide is given in the Hardware folder of the repository.

### One mirror or two?

Using two mirrors to steer a beam from one vector to another is taken as dogma for most applications. It is true that this is necessary for arbitrary output and input vectors, but that’s not the case here.

Lasers with identical beam heights and angle relative to the breadboard are used (assuming proper machining tolerances on the mounting block) and as a result one degree of freedom is removed. As such one does not need four adjusters to steer the beam into the fiber coupler, but rather three. This means a single three-adjuster mirror mount can be used and, as a result, six laser heads and steering optics can be squeezed into a single 450 mm x 300 mm footprint. This layout is given in the NicoLase 1500 description.

A more typical two mirror layout is used in the NicoLase 2400. This uses a pair of two-adjuster mirrors for each laser and fits four lasers into the 450 mm x 300 mm footprint. Using two mirrors does have more tolerance for lasers that might have unequal beam heights, such as when mixing Vortran and OBIS diode heads in a single unit. This layout also leaves a portion of the breadboard unused, meaning space is available for the second fiber coupler output path.

Alignment of a single-mirror system is only marginally more difficult than aligning the two mirror system. The single-mirror system is more stable. In both cases having three-adjuster mirrors is convenient to allow minor adjustments of the lateral beam position.

### One fiber or two?

In one application we combine TIRF excitation path with a laser scanner confocal-like path for extended imaging capability on the same chassis, sharing lasers, controllers, objectives, and camera. With products such as the Nikon stratum structure on the Ti series of microscopes having multiple excitation paths is reasonably straightforward. This approach could also be particularly advantageous for generating a pair of counter-propagating light sheets to reduce shadowing artefacts in light sheet microscopy applications [11]. The light paths are not going to be used simultaneously (though, in theory, they could be) so a means to selectively divide laser power between one of two exit ports is needed.

The method employed here uses a polarizing beamsplitter cube in conjunction with a half wave plate on a rotational mount. Linearly polarized incident light is divided between either the transmitted (P polarized) or reflected (S polarized) light paths depending on the angle of the incident polarization relative to the plane of the beamsplitter face. Rotating a half wave plate in front of the polarizing beamsplitter rotates the angle of polarization in the incident beam, controlling the division of laser power between the two exit light paths. This approach has the advantage of maintaining alignment between the two light paths while switching, as opposed to a flip mirror that may not reliably return to the same alignment each time. This enhancement requires that all lasers have a common polarization axis (nearly always within a few degrees of vertical for diode lasers) and corresponding quarter-wave plates or other depolarization optics at the fiber output, if the application requires.

Because of the tight space allowed between the final laser combiner mirror and the transmitted fiber output, a custom mount was designed (Fig 2). This mount holds a 12.5 mm polarizing beamsplitter cube (Thorlabs PBS122) and a 30 mm cage rotational mount (Thorlabs CRM1/M) with an achromatic half wave plate (Newport 10RP52-1). The mount was 3D printed and set of four 6 mm stainless steel rods are press-fit into the 3D printed part to hold the cage mount. Estimated cost for 3D printing *via* standard fused deposition modeling is $13. The mount is held on the breadboard with a standard 1” pedestal mount and at the proper beam height. A pair of half-inch mirrors on 25 mm pedestals steer the beam to a second fiber launch (due to the imprecision of the beamsplitter mount, two steering mirrors are necessary).

An optional second rotational mount with a linear polarizer (Newport 5511) can be placed upstream of the half wave plate to add an additional layer of polarization clean-up, though it is difficult to find a polarizer with transmission sufficiently broad to cover the wavelengths used here.

The overall coupling efficiency of the two NicoLase implementations are given in Tables 1 and 2. Each laser was set to a nominal power of 10 mW and the power measured at the far end of the output fiber. Coupling efficiencies are comparable between the two systems, falling between 31–68% for most wavelengths, being slightly higher in the 1500 design. The coupling of the 405 nm laser is confounded on the 2400 by the additional polarization optics which are rated to a shortest wavelength of 450 nm and 420 nm for the polarizer and beamsplitter, respectively. The power of the 405 nm line immediately downstream of the polarizer and upstream of the half wave plate is 3.48 mW (at 10 mW nominal from the laser), giving a coupling into the transmitted fiber of 58.91% and 10.06% into the reflected fiber. For the desired application the power at the 405 nm wavelength is small and can ultimately be compensated by the typically higher maximum output power of diodes of that wavelength relative to other wavelengths.

**Table 1:** Power throughput and coupling efficiencies for the NicoLase 1500 implementation.

NicoLase 1500 fiber couping			
Wavelength	Power at fiber input	Maximum fiber output power	Coupling efficiency
405 nm	10.03 mW	2.99 mW	29.81%
490 nm	9.55 mW	5.14 mW	53.82%
517 nm	9.15 mW	6.26 mW	68.42%
561 nm	8.41 mW	4.52 mW	53.75%
637 nm	8.50 mW	3.58 mW	42.12%

For these measurements all lasers were set to 10 mW nominal CW power.

**Table 2:** Power throughput and coupling efficiencies for the NicoLase 2400 implementation.

NicoLase 2400 fiber couping					
Wavelength	Power at beamsplitter	Transmitted fiber output power (max/min)	Reflected fiber output power (max/min)	Max coupling efficiency (trans/reflect)	Extinction ratio (trans/reflect)
405 nm	9.86 mW	2.050 mW/0.5 *μ*W	350 *μ*W/1.8 *μ*W	20.79%/3.55%	4,100:1/194:1
490 nm	9.75 mW	5.01 mW/2.2 *μ*W	5.40 mW/64.5 *μ*W	51.38%/55.38%	2,280:1/83.7:1
561 nm	9.16 mW	4.16 mW/0.4 *μ*W	4.69 mW/41.1 *μ*W	45.20%/51.20%	10,400:1/114:1
640 nm	5.30 mW	1.66 mW/0.5 *μ*W	1.44 mW/7.3 *μ*W	31.32%/27.17%	3,3200:1/197:1

For these measurements all lasers were set to 10 mW nominal CW power. Powers were tuned from minimum to maximum by rotating the half wave plate upstream of the polarizing beamsplitter.

In the 2400 implementation, the beam steering optics performed well in selectively driving power to a desired fiber output. Extinction of over 10,000:1 is possible through the transmitted path with rotation of the half wave plate. This value falls to 197:1 to 83.7:1 for the reflected path with 640 nm and 490 nm, respectively, which results in persistent bleed-through into that port. It’s a testament to the alignment capability that this light reaches the fiber in the first place, even with the imprecision in the printed mount. The bleed-through into the reflected output path is of little concern in the end as this emission can be hid behind a shutter in that light path. Laser output power can still be steered into one or the other (or both, if desired) light paths selectively without a large loss as would occur with, for example, a 50/50 beamsplitter in the same role, and still with fitting in the same 450 mm x 300 mm footprint.

Ideally the selection of fiber output could be accomplished remotely under computer control. Such an improvement could take the form of a motor-controlled rotation mount for the half-wave plate, which could then be addressed over a serial port or other controller. One could also take advantage of devices designed to selectively couple to multiple output fibers from a single input, such as the Leoni FiberSwitch. These devices provide rapid switching for comparable cost to the parts list given here ($1200).

## Controller

Trigger translation, especially with a time-variable component, requires some form of controller. A DAC board (such as the many options from National Instruments) is the “professional” choice, but are overkill for this application. A pair input channels and no more than eight output channels are needed. Timing precision, rise times, and trigger jitter on the microsecond or even tens of microseconds time scale is actually more than sufficient for most fluorescence microscopy applications (10 microseconds of jitter on a 10 ms exposure is a 0.1% variation). USB or serial control is convenient to communicate with the controller, as is low cost for the unit and the I/O interface.

Enter the Arduino Uno. Units cost $40 AUD ($25 USD) for an official product (with generic models under $10 AUD or $4 USD). Timing precision and programming space is sufficient, I/O interfaces are straightforward with an add-on shield, and a USB serial port comes standard. Bonuses are a large community of support and open-source, freely-available software for programming.

Another Arduino variant or another microcontroller can be swapped in if the need arises (*e.g*. if the application requires more analog I/O ports, or a 32-bit timer, or additional serial ports) but that’s beyond the scope of this project. As long as the unit can take a master trigger in, output the appropriate pulses, and communicate over a serial port, the exact product can be chosen to meet the needs of the application.

Throughout the discussion on triggering, the catch-all term ‘lasers’ or ‘laser outputs’ for the end target for TTL outputs of the controller is used. This same controller and software can be used with any equipment receiving a TTL input pulse that needs to be synced to an external clock signal. For example, a LED illuminator for transmitted light is commonly used with the controller output while receiving the camera fire signal as the external clock. The controller, shield, and software ultimately can be adapted to a number of applications beyond the presented one where a low-cost means to synchronize or sequence a small number of pieces of equipment through TTL signals is required.

**Fig 3:**
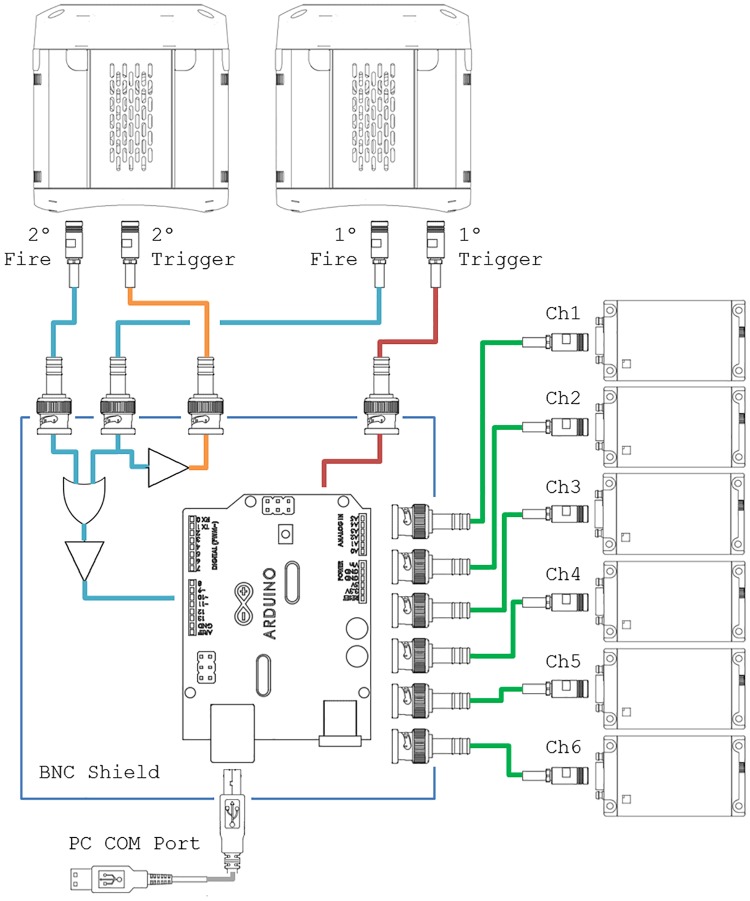
Circuit diagram of the NicoLase Arduino controller. A master fire signals (blue) pass through an OR gate before a 5v buffer and triggering the Arduino Uno. The master fire of the primary camera can also be used to trigger the secondary camera (orange). The Arduino detects incoming trigger signals and, depending on the programmed sequence, sends outgoing signals to one or more of the six output channels (green). Additionally the Arduino can trigger the primary camera directly (red).

Interfacing with the trigger pulses in and out is accomplished with a custom shield. A schematic of the circuit appears in Fig 3. Additionally EAGLE schematic files appear in the ShieldPCB folder of the GitHub repository. The PCB includes 10 BNC ports (two camera inputs, two camera outputs, and six laser outputs), a OR gate, 3v3 to 5v level shifter, and pushbutton, plus a few pull-down and limiting resistors. The shield mounts directly on top of the Arduino. The Arduino and shield is powered over the USB port and does not require a separate power supply.

Two software packages are provided in the repository. In the Basic Controller, the synchronization signal is provided by an external source; this is typically the primary system camera. An Advanced System Controller optionally allows the Arduino to be the master clock for the system. Downstream triggering and sequencing operates identically for each configuration mode.

With the Arduino listening for external clock pulses all connected devices are synchronized to a chosen source. This synchronization source is free to vary in timing or be aperiodic with no change in operation of the controller. The Arduino as the master clock provides additional functionality as a standalone instrument controller for up to seven TTL devices (one triggering on each clock pulse and six on a programmable sequence) which we hope may be useful in other scientific settings. The Arduino as as the clock source also allows relieves other devices of this duty and may reduce unwanted overhead or timing delays from those components.

Within the controller each acquisition frame is synchronized by the 1°Fire signal being driven high; this is either by the camera’s internal triggering or through the 1°Trigger input from the controller itself. This 1°Fire camera input drives the 2°Trigger output high as well as one input of the 3v3 OR gate. This allows the primary camera to directly trigger a frame capture on the secondary camera for dual-camera acquisitions (on single-camera systems the secondary camera ports are left empty). The other OR gate input is the 2°Fire signal, meaning either camera can be used singly to trigger the Arduino without reprogramming or reconnecting the controller inputs. The OR gate output connects through a 3v3 to 5v level shifter to ensure that the pulse adequately triggers 5v input on the Arduino. There the 5v pulse connects to interrupt digital pin 2 of the Arduino. Arduino digital pin 3 connects to pushbutton on the PCB which can also be used to iterate laser sequence for testing in place of a camera trigger. Digital pin 4 goes through a current-limiting resistor to the 1°Trigger output, through which the Arduino is used to trigger the primary camera. Through this pin the Arduino can act as the master system clock or to initiate an acquisition at a pre-set time, depending on configuration mode.

All TTL outputs occur on Analog Out register of Arduino Uno. Outputs sharing a common register means all can be set simultaneously in the Arduino software without having to go through the digitalWrite() function. This allows faster triggering and all outputs are set with the same delay/jitter rather than a variable delay depending on which pin on the register they lie. Outgoing pulses are all 5v TTL levels. Incoming signals are anticipated to be 3v3 TTL signals with the pins tolerant to 5v signals.

A bill of materials included in the repository for the parts necessary to populate the shield. Surface mount components are used to fit into the space available. All should be common components available from any major electronics supplier.

## Software

Software comprises of two parts—controller software running on the Arduino and interface software for the PC. Only the controller software is strictly necessary for triggering. Examples of PC software are given which can be expanded to work with your particular system.

### Controller sketch

The Arduino sketch accomplishes two primary needs—external communication and sequence programming via serial port, and triggering a programmed output sequence in response to a trigger pulse. The output sequence is an N x 6 binary array, with each line corresponding to the output configuration that will be adopted on a input trigger pulse. A change in the trigger pin state triggers an interrupt on the Arduino. A rising edge causes the outputs (CH1 through CH6) to adopt the current line of the output sequence. A falling edge on the trigger pulse drives all outputs low and iterates the sequence program counter. Any given line of the sequence can be repeated any number of times (up to 2147483647 times per line) before proceeding to the next line if programmed by the user. Once the end of the sequence is reached the Arduino goes back to the start and repeats indefinitely.

Serial commands are via the USB connection to the control PC. Upon startup, the Arduino adopts the default output sequence (*i.e*. all outputs high when the fire signal is high). It also initializes communication with the instrument PC, listening for commands coming over the serial port. A detailed description of the possible serial commands is given in the documentation in the Arduino Sketch folder of the repository. Each command is a capital letter character specifying the command type, in some cases a space followed by one or more digits, and always terminated by a newline character. For example, the command ‘B 000111\n’ will append an entry to the current output sequence where channels 1–3 (CH1, CH2, and CH3) are high and channels 4–6 (CH4, CH5, and CH6) low. A glossary of supported serial commands is given in the repository.

Alternate functions are available in the Basic Controller mode for what is called a pre- or post-acquisition command. These are single output patterns that can be triggered by a serial command independent of a trigger input. Each has a time associated for how long the outputs will be held high. The pre-acquisition sequence has an additional delay after which an optional pulse will be send to the 1°Trigger pin. This means the user can, for example, have an acquisition sequence of a 488 nm laser and 561 nm laser firing on alternating camera frames (the ‘acquisition sequence’). Before that sequence is reached, however, a pre-acquisition sequence of a 405 nm laser is fired with no camera acquisition for 1000 ms, followed by a 500 ms delay, and then the actual acquisition begun by the Arduino sending a pulse to the master camera. A more detailed description of the triggering and associated controller functions is given in the Triggering section,.

Within the Advanced System Controller configuration, the controller can be set to either listen for incoming clock pulses, as in the Basic Controller mode, or as the master clock for the system. The associated sketch is again using serial commands, with additional parameters for timing of each frame in the sequence and for the sequence loop. Each frame in the loop can be iterated an arbitrary number of times providing fine control over the acquisition protocol. Readers are referred to the repository for detailed descriptions of controller programming.

### PC software

Software on the PC side is used to alter the sequence on the Arduino controller. Any program capable of communicating through a serial port can be used. As this is a common means for communication with microscopy peripherals, it is straightforward to include the controller as a device in most instrument control softwares. Micro-manager [12] was the focus of much of the testing for its ever-increasing capabilities and as the leading freely-available microscope control platform. The Arduino is added to the Micro-manager configuration as a serial port device. Micro-manager then passes along strings to the Arduino at the beginning of the experiment (or during, if desired) which then sets the Arduino sequence configuration and listens for incoming camera trigger pulses or drives the acquisition timing directly.

Example Micro-manager Beanshell scripts to communicate output sequence settings are given as well as a MATLAB (MathWorks) GUI for easily uploading arbitrary sequences through the Basic Controller configuration. The Advanced System Controller software includes a Micro-manager plug-in with GUI interface for conveniently selecting output sequences, setting timings, and including cameras in individual acquisition steps. This plug-in further assembles acquired images into the proper multi-dimensional stack based on user-defined camera and sequence settings.

### Triggering

**Fig 4:**
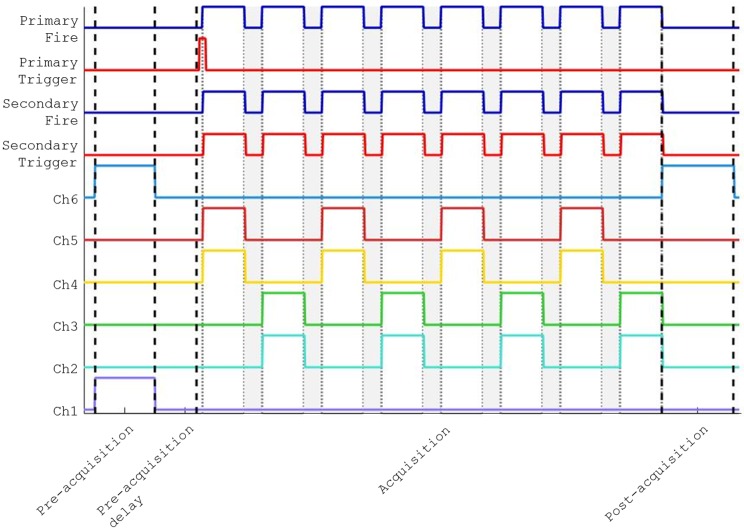
Triggering diagram with NicoLase controller. Here input signals are blue (Primary In and Secondary In), outputs to cameras in red (Primary Out and Secondary Out), and outputs to lasers in wavelength-approximate colors (Ch6–Ch1). Trigger event stages are separated by dashed black lines with acquisition frames in gray blocks with dotted borders.

A diagram of the triggering sequence under the external clock mode is given in Fig 4. The progression of trigger signals for this experiment of 8 imaging frames is as follows:

1. Serial command to Arduino starts Pre-acquisition sequence with Ch1 and Ch6 high (*i.e*. 405 nm laser and blue transmitted LED)
2. After defined illumination time, Pre-acquisition ends and Pre-acquisition delay begins
3. After defined pre-acquisition delay time, a pulse is sent to the 1°Trigger output
4. The Primary camera begins acquiring frames (characteristics defined through camera software). Each is associated with an input pulse at the 1°Fire pin
5. Each incoming 1°Fire pulse results in an output 2°Trigger pulse and output pulses to the user-specified CHX output pins. Here alternating sequences of [Ch5 + Ch4] and [Ch3 + Ch2] are shown to illustrate the programmable sequencing of these pins.
6. Each 2°Trigger pulse triggers the secondary camera, which in turn results in an incoming 2°Fire pulse (ignored in 2-camera acquisitions)
7. Falling edge of 1°Fire pulse drives CHX outputs low and Arduino iterates to next line of sequence
8. After acquisition is complete, serial command to Arduino starts Post-acquisition sequence (here Ch6, blue transmitted LED high) for user-defined time

The triggering signals in the Arduino-driven master clock mode differ in that an outgoing pulse at the 1°Trigger output is sent every frame rather than once at the start of the acquisition (step 3). The Primary camera additionally waits for an incoming pulse before acquiring each frame rather than being triggered by software or an internal clock (step 4).

In practice the separation between the 1°Fire and 2°Fire pulses, representing the round-trip time of the signal through the secondary camera, is on the order of single microseconds. As a result the acquisition times on the two cameras are treated as simultaneous. Single-camera systems can work on either input, though using 1°Fire input is recommended. This experimental framework covers a large number of potential applications by expanding the triggering and sequencing options. This should provide a scheme to control a number of imaging instrumentation devices and hopefully aids other researchers in creating their own application-specific designs.

**Fig 5:**
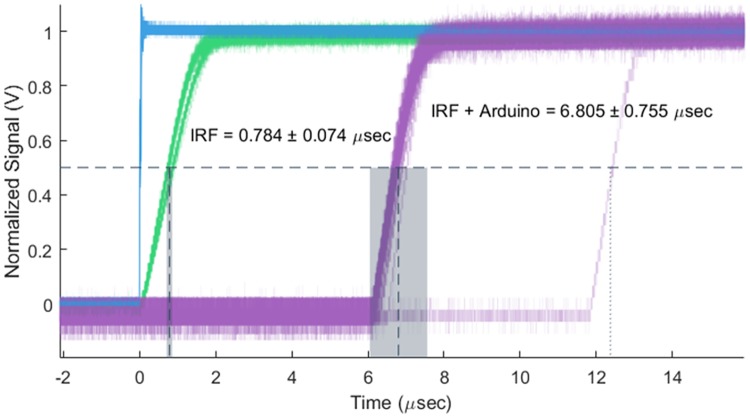
Timing performance of NicoLase controller. A 10 Hz square wave signal from a function generator (blue) provides a trigger signal to the NicoLase Arduino controller (‘IRF + Arduino’, purple) or to the diode laser directly (‘IRF’, green). Mean rise times to half amplitude of normalized signal (dashed lines with standard deviations in gray) are as indicated. Occasional (1 of 57 here) triggers are caught on a second clock cycle by the controller, with a maximum observed delay between incoming trigger and laser firing of 12.38 *μ*s.

To evaluate the timing performance of the control system we used an arbitrary function generator (AFG) to drive the controller triggering while monitoring the laser intensity at the output of the single-mode fiber with a photodetector (Thorlabs DET210) and oscilloscope (Tektronix MDO3012, with AFG module). The AFG generated a 5v square wave at 10 Hz input either directly to the laser diode or the Primary Fire pin on the NicoLase controller shield. Fig 5 shows the results of these measurements using a 561 nm Vortran Stradus diode. The instrument response function of the system, including the rise time of the laser and photodetector, is 0.754 ± 0.074 *μ*s (mean ± standard deviation, N = 26). With the AFG driving the Arduino controller, this time is increased to 6.805 ± 0.755 *μ*s (N = 57). Occasionally (1 of 57 times here) the light pulse would be detected at a later time point (12.380 *μ*s), possibly indicating the trigger falling between clock cycles on the Arduino. No outgoing pulses were detected later than this. Even with this amount of jitter, the delay is negligibly short on the timescale of a typical fluorescence microscopy imaging experiment. Assuming a 30 ms exposure time, the longest observed rise time from the incoming trigger would give a non-exposed period 0.041% of the total frame time.

## Conclusion

The motivation and implementation of two generations of a diode laser combiner, fiber launch, and controller. The designs utilize commonly-available components to yield a low-cost solution for multi-wavelength imaging. Software for syncing multiple camera triggers with programmable laser sequencing is included. All design files, machine shop drawings, parts lists, and software are openly available for users to clone, modify, and share through the project GitHub repository. Together this provides an open platform for an aspect of fluorescence microscopy previously left to ad-hoc or closed-source commercial instruments.
